# Age‐wise examination of the association of obesity based on body mass index and waist circumference with metabolic diseases in comprehensive health checkup participants

**DOI:** 10.1002/osp4.746

**Published:** 2024-03-18

**Authors:** Yuiko Yamamoto, Kentaro Ikeue, Megumi Kanasaki, Hajime Yamakage, Noriko Satoh‐Asahara, Izuru Masuda, Kojiro Ishii

**Affiliations:** ^1^ Graduate School of Health and Sports Science Doshisha University Kyotanabe Japan; ^2^ Department of Endocrinology, Metabolism, and Hypertension Research Clinical Research Institute National Hospital Organization Kyoto Medical Center Kyoto Japan; ^3^ Medical Examination Center Takeda Hospital Kyoto Japan; ^4^ Department of Molecular Medicine and Metabolism Research Institute of Environmental Medicine Nagoya University Nagoya Japan; ^5^ Faculty of Health and Sports Science Doshisha University Kyotanabe Japan

**Keywords:** health checkup, metabolic diseases, obesity, older adults, visceral fat

## Abstract

**Aim:**

Body mass index and waist circumference are used for obesity diagnosis and screening of visceral fat; however, their evidence in older adults is insufficient. This study investigated the age‐specific association of body mass index and waist circumference with metabolic diseases, assessing their applicability as diagnostic criteria for individuals aged ≥65 years.

**Methods:**

Analysis included 46,324 individuals aged ≥18 years, categorized into five age groups: 18–44, 45–54, 55–64, 65–74, and ≥75 years. Logistic regression analyses identified associations between obesity and metabolic diseases, stratified by age and sex.

**Results:**

Men with obesity based on body mass index had a significantly high risk of hypertension, diabetes mellitus, and dyslipidemia across all age groups (all, *p* < 0.05). Obesity based on waist circumference was significantly positively associated with all metabolic diseases (all, *p* < 0.05). Women with obesity based on body mass index and waist circumference had a significantly high risk of all metabolic diseases across all age groups (all, *p* < 0.05), except for diabetes mellitus in individuals aged ≥75 years.

**Conclusions:**

Participants with obesity based on body mass index and waist circumference exhibited a high risk of hypertension, diabetes mellitus, and dyslipidemia among those aged 18–74 years and men aged ≥75 years. This study contributes to the early prevention and control of metabolic diseases.

## 1 INTRODUCTION

The Japan Society for the Study of Obesity defines obesity as a body mass index (BMI) ≥25 kg/m^2^. Additionally, visceral fat accumulation in screening is defined as a waist circumference (WC) ≥85 cm in men and ≥90 cm in women.^1^, ^2^ However, the examination of these criteria in older adults aged ≥65 years was not conducted adequately.^2^, ^3^, ^4^ A previous study, which indicated that a BMI of 22 kg/m^2^ was associated with the lowest morbidity, only included participants aged <60 years.^4^ Notably, BMI may not accurately reflect body fat in older adults due to factors such as decreased height and edema^2^, ^3^ Furthermore, visceral fat mass tends to increase with age but not body weight.^5^ Therefore, the question remains whether BMI and WC can assess the risk of metabolic diseases caused by obesity or not in older adults.

In contrast, Japan has the system “Specific Health Checkups and Specific Health Guidance” for adults aged 40–74 years.^6^ This system focuses on prevention and early detection of metabolic syndrome.^6^ In this system, BMI and WC are used as obesity‐related items for screening individuals in whom the health guidance applies, and these criteria are the same for individuals aged 40–74 years.^6^

Additionally, in 2013, the Japan Gerontological Society and the Japan Geriatrics Society launched a joint committee on the definition and classification of older adults.^7^, ^8^ The data by this joint committee on the physical and psychological health of older adults indicated a delay of approximately 5–10 years in age‐related changes in physical and psychological functions, compared with the findings from 10 to 20 years ago.^7^, ^8^ The age‐specific rate of estimated patients with most chronic medical conditions in older adults has also decreased annually.^8^, ^9^ Therefore, this joint committee proposed classifying those aged 65–74 years as “pre‐old” and those aged ≥75 years as “old”.^7^, ^8^ However, there is a lack of data on obesity index related to the classification of older adults, compared with the prevalence of diseases, physical function, and psychophysiological indices.

From the above, the examination whether the currently used obesity index is associated with metabolic diseases in older adults aged 65–74 or ≥75 years, as in other age groups, is necessary. Therefore, this study aimed to clarify the association of BMI and WC with metabolic diseases across different age groups and assess the applicability of these diagnosis criteria in individuals aged ≥65 years.

## 2 PARTICIPANTS AND METHODS

### 2.1 Study participants

In this study, the data from individuals who underwent health checkups at the Takeda Hospital Medical Examination Center (Kyoto, Japan) between April 2011 and June 2022, were used. Participants underwent an annual basic health checkup that is required by law for employees, Specific Health Checkups for adults aged 40–74 years, or a comprehensive health checkup voluntarily. Most of the participants usually had a good health condition to work or to come for health checkups by themselves. Of a total of 101,507 participants, 46,324 individuals (21,628 men and 24,696 women) aged ≥18 years with complete data on age, sex, height, weight, WC, blood pressure, glucose metabolism index, lipid metabolism index, and medications were included in the analysis and classified into five age groups: 18–44, 45–54, 55–64, 65–74, and ≥75 years. The older adults were classified into 65–74 and ≥75 years because Specific Health Checkups are for individuals aged <75 years, and also the evidence in older adults aged ≥75 years is particularly insufficient.^3^ Additionally, the prevalence of metabolic diseases is less common markedly before middle‐age^10^; hence, those aged <45 years were put into one group. Individuals aged 45–64 years applied the classification by the age of 10 years to unify with older adults aged 65–74 years.

This study was approved by the Ethics Committee of Takeda Hospital Group (Approval number: 2028), the Ethics Committee for Human Research at National Hospital Organization Kyoto Medical Center (Approval number: 20–093), and the Doshisha University Research Ethics Committee (Approval number: 20052), and conformed to the provisions of the Declaration of Helsinki (as revised in Brazil 2013). Informed consent was obtained in an opt‐out format by posting a written explanation at Takeda Hospital, Medical Examination. Those who rejected participation were excluded from the study.

### 2.2 Definition of obesity and metabolic diseases

BMI was calculated by dividing the body weight by the square of the height. WC was measured at the navel in a standing position. Following the diagnosis criteria outlined by the Japan Society for the Study of Obesity, obesity was defined as having a BMI ≥25 kg/m^2^ or WC ≥85 cm in men and ≥90 cm in women, respectively.^1^, ^2^ Metabolic diseases were identified according to the guidelines for each disease. Hypertension was defined as systolic blood pressure ≥140 mmHg, diastolic blood pressure ≥90 mmHg, or the use of antihypertensive medications.^11^ Diabetes mellitus was defined as fasting blood glucose ≥126 mg/dL, glycosylated hemoglobin levels ≥6.5%, or the use of antidiabetic medications.^12^ Dyslipidemia was defined as low‐density lipoprotein cholesterol ≥140 mg/dL, high‐density lipoprotein cholesterol <40 mg/dL, triglyceride levels ≥150 mg/dL, or the use of lipid‐lowering medications.^13^

### 2.3 Statistical analysis

The characteristics of the study participants were presented as mean ± standard deviation or number (percentage). To clarify the association between BMI and WC and metabolic diseases, logistic regression analyses were performed by age and sex. The presence or absence of metabolic disease (presence of each disease: 1, absence of each disease: 0) as the objective variable, and the presence or absence of obesity based on each index (individuals without obesity as reference) was used as the explanatory variable. An unadjusted model was performed as Model 1, and age was put into the moderator variable in Model 2. All statistical analyses were conducted using IBM SPSS Statistics 29.0 (Japan IBM). A *p*‐value <0.05 was considered statistically significant.

## 3 RESULTS

### 3.1 Characteristics of the study participants

Tables 1 and 2 present the characteristics of men and women, respectively, among the study participants. BMI and WC were higher in men than in women (23.7 ± 3.4 kg/m^2^ vs. 21.8 ± 3.6 kg/m^2^ and 84.2 ± 9.2 cm vs. 77.1 ± 9.4 cm, respectively), with more men than women having obesity based on BMI and WC (BMI: 30.0% vs. 15.5% and WC: 42.5% vs. 9.3%). Of all the metabolic diseases in both men and women, respectively, dyslipidemia had the highest prevalence (56.4% and 39.8%), followed by hypertension (37.8% and 20.7%) and diabetes mellitus (10.0% and 3.3%).

**TABLE 1:** Characteristics of the male study participants.

	Total	18–44 years	45–54 years	55–64 years	65–74 years	≥75 years
	(*n* = 21,628)	(*n* = 6362)	(*n* = 6532)	(*n* = 5228)	(*n* = 2707)	(*n* = 799)
Age (years)	52.0 ± 12.3	38.0 ± 4.7	49.3 ± 2.8	59.3 ± 2.8	69.1 ± 3.0	79.5 ± 3.8
Height (cm)	170.9 ± 6.1	172.6 ± 5.9	171.9 ± 5.7	170.4 ± 5.7	167.7 ± 5.6	164.6 ± 5.8
Weight (kg)	69.3 ± 11.2	69.8 ± 11.7	71.1 ± 11.8	69.5 ± 10.4	65.4 ± 9.0	62.5 ± 8.8
BMI (kg/m^2^)	23.7 ± 3.4	23.4 ± 3.6	24.0 ± 3.6	23.9 ± 3.2	23.3 ± 2.9	23.0 ± 2.8
BMI ≥ 25 kg/m^2^	6490 (30.0)	1741 (27.4)	2213 (33.9)	1680 (32.1)	674 (24.9)	182 (22.8)
WC (cm)	84.2 ± 9.2	82.4 ± 9.6	84.7 ± 9.5	85.4 ± 8.6	84.5 ± 8.0	84.6 ± 8.2
WC ≥ 85 cm	9198 (42.5)	2211 (34.8)	2879 (44.1)	2496 (47.8)	1238 (45.7)	374 (46.8)
Hypertension	8175 (37.8)	928 (14.6)	2228 (34.1)	2768 (52.9)	1678 (62.0)	573 (71.7)
Diabetes mellitus	2170 (10.0)	133 (2.1)	504 (7.7)	794 (15.2)	539 (19.9)	200 (25.0)
Dyslipidemia	12,208 (56.4)	2838 (44.6)	3830 (58.6)	3306 (63.2)	1749 (64.6)	485 (60.7)

*Note*: Values are presented as mean ± standard deviation or number (percentage).

Abbreviations: BMI, body mass index; WC, waist circumference.

**TABLE 2:** Characteristics of the female study participants.

	Total	18–44 years	45–54 years	55–64 years	65–74 years	≥75 years
	(*n* = 24,696)	(*n* = 8989)	(*n* = 7432)	(*n* = 5306)	(*n* = 2338)	(*n* = 631)
Age (years)	49.3 ± 12.7	36.2 ± 6.0	49.4 ± 2.8	59.0 ± 2.8	69.1 ± 2.9	79.2 ± 3.4
Height (cm)	158.0 ± 5.6	159.2 ± 5.3	158.9 ± 5.3	157.2 ± 5.2	154.3 ± 5.2	151.0 ± 5.2
Weight (kg)	54.3 ± 9.4	53.9 ± 9.3	55.6 ± 9.9	54.4 ± 9.4	52.5 ± 8.0	50.9 ± 7.6
BMI (kg/m^2^)	21.8 ± 3.6	21.2 ± 3.4	22.0 ± 3.7	22.0 ± 3.7	22.1 ± 3.3	22.3 ± 3.2
BMI ≥ 25 kg/m^2^	3837 (15.5)	1008 (11.2)	1316 (17.7)	971 (18.3)	412 (17.6)	130 (20.6)
WC (cm)	77.1 ± 9.4	74.3 ± 8.6	77.6 ± 9.6	79.1 ± 9.6	80.1 ± 8.8	81.9 ± 8.6
WC ≥ 90 cm	2299 (9.3)	475 (5.3)	745 (10.0)	658 (12.4)	304 (13.0)	117 (18.5)
Hypertension	5107 (20.7)	394 (4.4)	1344 (18.1)	1733 (32.7)	1171 (50.1)	465 (73.7)
Diabetes mellitus	825 (3.3)	57 (0.6)	182 (2.4)	273 (5.1)	220 (9.4)	93 (14.7)
Dyslipidemia	9838 (39.8)	1478 (16.4)	2862 (38.5)	3386 (63.8)	1665 (71.2)	447 (70.8)

*Note*: Values are presented as mean ± standard deviation or number (percentage).

Abbreviations: BMI, body mass index; WC, waist circumference.

### 3.2 Association between obesity and metabolic diseases

Tables 3 and 4 show the results of logistic regression analyses for men and women, respectively. Among men, in both the unadjusted and age‐adjusted models, participants with obesity (BMI ≥25 kg/m^2^) had a significantly higher risk of hypertension, diabetes mellitus, and dyslipidemia (all, *p* < 0.05), compared with that of those without obesity across all age groups. Similarly, obesity based on a WC of ≥85 cm was significantly positively associated with all metabolic diseases (all, *p* < 0.05).

**TABLE 3:** Odds ratios of obesity for metabolic diseases in men.

	Model 1^a^			Model 2^b^		
	OR	95% CI	*p*‐value	OR	95% CI	*p*‐value
Hypertension						
BMI^c^						
18–44 years	4.75	4.11–5.49	<0.001	4.64	4.01–5.38	<0.001
45–54 years	3.63	3.25–4.05	<0.001	3.73	3.34–4.16	<0.001
55–64 years	2.87	2.53–3.24	<0.001	2.92	2.58–3.30	<0.001
65–74 years	2.87	2.34–3.52	<0.001	2.96	2.41–3.64	<0.001
≥75 years	5.79	3.33–10.07	<0.001	6.03	3.46–10.51	<0.001
WC^c^						
18–44 years	4.32	3.73–5.00	<0.001	4.13	3.56–4.79	<0.001
45–54 years	3.33	2.99–3.70	<0.001	3.34	3.00–3.72	<0.001
55–64 years	2.57	2.30–2.87	<0.001	2.57	2.30–2.87	<0.001
65–74 years	2.48	2.11–2.92	<0.001	2.50	2.13–2.95	<0.001
≥75 years	2.74	1.97–3.81	<0.001	2.82	2.02–3.93	<0.001
Diabetes mellitus						
BMI^c^						
18–44 years	10.06	6.64–15.24	<0.001	9.55	6.30–14.48	<0.001
45–54 years	5.24	4.30–6.39	<0.001	5.32	4.36–6.50	<0.001
55–64 years	2.61	2.24–3.05	<0.001	2.66	2.28–3.10	<0.001
65–74 years	1.89	1.55–2.32	<0.001	1.93	1.57–2.37	<0.001
≥75 years	1.46	1.01–2.11	0.043	1.48	1.03–2.14	0.036
WC^c^						
18–44 years	8.48	5.47–13.14	<0.001	7.92	5.11–12.29	<0.001
45–54 years	5.37	4.31–6.69	<0.001	5.32	4.27–6.64	<0.001
55–64 years	2.61	2.22–3.06	<0.001	2.60	2.21–3.05	<0.001
65–74 years	1.65	1.36–1.99	<0.001	1.65	1.37–2.00	<0.001
≥75 years	1.51	1.09–2.08	0.012	1.52	1.10–2.10	0.010
Dyslipidemia						
BMI^c^						
18–44 years	3.51	3.13–3.95	<0.001	3.45	3.07–3.88	<0.001
45–54 years	2.8	2.54–3.19	<0.001	2.85	2.55–3.19	<0.001
55–64 years	2.49	2.19–2.84	<0.001	2.49	2.18–2.84	<0.001
65–74 years	1.99	1.64–2.43	<0.001	1.98	1.63–2.42	<0.001
≥75 years	1.91	1.33–2.74	<0.001	1.89	1.32–2.72	<0.001
WC^c^						
18–44 years	3.47	3.12–3.87	<0.001	3.36	3.02–3.75	<0.001
45–54 years	2.71	2.45–3.01	<0.001	2.71	2.44–3.00	<0.001
55–64 years	2.35	2.09–2.64	<0.001	2.35	2.09–2.64	<0.001
65–74 years	1.84	1.57–2.16	<0.001	1.84	1.56–2.16	<0.001
≥75 years	1.98	1.48–2.65	<0.001	1.97	1.47–2.64	<0.001

Abbreviations: BMI, body mass index; CI, confidence interval; OR, odds ratio; WC, waist circumference.

^a^ Model 1, unadjusted.

^b^ Model 2, adjusted for age.

^c^ Individuals without obesity based on each obesity index were set as a reference group.

**TABLE 4:** Odds ratios of obesity for metabolic diseases in women.

	Model 1^a^			Model 2^b^		
	OR	95% CI	*p*‐value	OR	95% CI	*p*‐value
Hypertension						
BMI^c^						
18–44 years	7.36	5.9–9.07	<0.001	6.57	5.30–8.15	<0.001
45–54 years	4.45	3.90–5.08	<0.001	4.52	3.95–5.17	<0.001
55–64 years	3.67	3.18–4.24	<0.001	3.71	3.21–4.29	<0.001
65–74 years	3.11	2.46–3.93	<0.001	3.15	2.49–3.99	<0.001
≥75 years	2.26	1.35–3.78	0.002	2.40	1.43–4.03	<0.001
WC^c^						
18–44 years	9.72	7.64–12.36	<0.001	8.48	6.62–10.85	<0.001
45–54 years	5.79	4.94–6.78	<0.001	5.69	4.85–6.68	<0.001
55–64 years	3.83	3.23–4.53	<0.001	3.83	3.23–4.54	<0.001
65–74 years	3.15	2.41–4.13	<0.001	3.25	2.48–4.26	<0.001
≥75 years	2.40	1.39–4.16	0.002	2.43	1.40–4.22	0.002
Diabetes mellitus						
BMI^c^						
18–44 years	23.09	12.76–41.7	<0.001	21.04	11.58–38.20	<0.001
45–54 years	9.80	7.17–13.40	<0.001	9.80	7.16–13.41	<0.001
55–64 years	5.07	3.96–6.50	<0.001	5.11	3.98–6.55	<0.001
65–74 years	2.61	1.92–3.53	<0.001	2.60	1.92–3.53	<0.001
≥75 years	1.42	0.85–2.36	0.181	1.45	0.87–2.43	0.154
WC^c^						
18–44 years	18.33	10.81–31.07	<0.001	16.25	9.53–27.70	<0.001
45–54 years	10.83	8.01–14.64	<0.001	10.40	7.68–14.08	<0.001
55–64 years	4.89	3.77–6.34	<0.001	4.86	3.75–6.31	<0.001
65–74 years	2.80	2.02–3.88	<0.001	2.83	2.04–3.94	<0.001
≥75 years	1.25	0.73–2.14	0.427	1.24	0.72–2.14	0.429
Dyslipidemia						
BMI^c^						
18–44 years	4.80	4.17–5.52	<0.001	4.53	3.93–5.22	<0.001
45–54 years	2.97	2.62–3.35	<0.001	3.04	2.68–3.44	<0.001
55–64 years	2.25	1.91–2.65	<0.001	2.25	1.92–2.65	<0.001
65–74 years	2.41	1.82–3.19	<0.001	2.41	1.82–3.19	<0.001
≥75 years	1.84	1.15–2.95	0.011	1.80	1.12–2.88	0.015
WC^c^						
18–44 years	5.62	4.65–6.80	<0.001	5.22	4.31–6.32	<0.001
45–54 years	3.11	2.66–3.64	<0.001	3.04	2.59–3.57	<0.001
55–64 years	1.90	1.57–2.29	<0.001	1.89	1.57–2.28	<0.001
65–74 years	2.72	1.95–3.81	<0.001	2.74	1.96–3.83	<0.001
≥75 years	2.12	1.28–3.53	0.004	2.14	1.29–3.56	0.003

Abbreviations: BMI, body mass index; CI, confidence interval; OR, odds ratio; WC, waist circumference.

^a^ Model 1, unadjusted.

^b^ Model 2, adjusted for age.

^c^ Individuals without obesity based on each obesity index were set as a reference group.

Among women, in both the unadjusted and age‐adjusted models, participants with obesity (BMI ≥25 kg/m^2^) had a significantly high risk of hypertension, diabetes mellitus, and dyslipidemia (all, *p* < 0.05), compared with those without obesity in all age groups, except for individuals aged ≥75 years. Similarly, obesity based on a WC of ≥90 cm was significantly positively associated with all metabolic diseases in all age groups (all, *p* < 0.05), except for individuals aged ≥75 years. In contrast, there was a significant association between obesity and hypertension (*p* < 0.05) and dyslipidemia (*p* < 0.05), but no association between obesity and diabetes mellitus in individuals aged ≥75 years, whether assessed by BMI or WC.

## 4 DISCUSSION

In men, obesity based on both BMI and WC was significantly positively associated with hypertension, diabetes mellitus, and dyslipidemia across all age groups. However, in women, there was no association between obesity, based on BMI and WC, and diabetes mellitus in individuals aged ≥75 years.

The current criteria for diagnosing obesity were established based on previous studies that reported that the incidence rates of hypertension, hyperglycemia, and hyperlipidemia were significantly increased at a BMI of 24–25.9 kg/m^2^, using a BMI of 20–23.9 kg/m^2^ as reference.^14^ Moreover, the average number of complications of hypertension, diabetes mellitus, and dyslipidemia exceeded 1.0 at a BMI of 25 kg/m^2^ and visceral fat area of 100 cm^2^, and these values were considered to indicate the best sensitivity and specificity for detecting individuals with two or more of these diseases.^1^ Therefore, a WC of ≥85 cm in men and ≥90 cm in women has become the screening criteria for visceral fat accumulation.^1^, ^2^ Studies involving participants aged 18–85 years in both Japan and the United States have consistently shown that those with a BMI indicating overweight and obesity, exhibited a higher risk of hypertension, diabetes mellitus, and dyslipidemia, compared to that of individuals with a normal BMI.^15^ Moreover, individuals with WC measurements of ≥85 cm in men and ≥90 cm in women exhibited a higher risk of developing diabetes mellitus^16^ and hypertension^17^ than that of those without such WC measurements. These previous findings align with the results of the present study in that obesity based on BMI and WC was associated with a high risk of metabolic diseases.

The study that included 63,180 Chinese and American participants by Zhou et al. indicated that a significant association between obesity and diabetes mellitus was observed in those aged ≥75 years as well.^18^ The present study in Japanese participants showed no such association in women aged ≥75 years; hence, these results are inconsistent. However, stratified analysis by region revealed that individuals with obesity aged ≥75 years had a high risk of diabetes mellitus in the US but not in China.^18^ Therefore, the inconsistency could be mainly due to ethnicity.^19^, ^20^ Originally, Japanese people have a lower insulin secretion than Caucasians.^19^ On the other hand, the prevalence of obesity is less common in Asia, including Japan.^20^

Obesity increases the synthesis and secretion of inflammatory cytokines, such as TNF‐α and free fatty acids, and oxidative stress.^21^, ^22^ These factors induce systemic inflammation, insulin resistance, and metabolic disorders, leading to metabolic diseases.^22^ In older adults, changes in body composition^5^, ^23^ and a decrease in height^24^ can occur. Consequently, BMI may not accurately reflect body fat in older adults.^2^, ^3^, ^25^ Additionally, the existence of an “obesity paradox” has been reported in older adults, in which the mortality risk is lower or remains the same for those who are overweight or have obesity according to BMI.^26^ However, as shown in the joint committee on the definition and classification of older people by the Japan Gerontological Society and the Japan Geriatrics Society, physical functions have improved in recent years by approximately 10 years.^7^, ^8^ Additionally, the health condition in those aged ≥65 years has been getting better and the age of onset of disease has been delayed in Japan.^9^ A study involving Japanese individuals aged 65 years has also reported that obesity was associated with the onset risk of metabolic diseases.^27^ Therefore, obesity based on BMI and WC is likely to be significantly associated with metabolic diseases in those aged 65–74 years as well as in those aged <65 years.

Metabolic diseases may be affected by other factors in individuals aged ≥75 years. For instance, skeletal muscle mass decreases with age,^28^ and the prevalence of sarcopenia increases.^29^, ^30^ A recent study has shown that a group with lower appendicular skeletal muscle mass per body weight had a higher risk of diabetes mellitus than those with higher muscle mass.^31^ Additionally, older adults with diabetes mellitus lose more appendicular lean mass,^32^, ^33^ trunk fat mass,^32^, ^33^ and total body mass^33^ than do those without diabetes. Therefore, in the present study, older women aged ≥75 years with diabetes mellitus might have already lost their body weight, fat mass, and lean mass. The quantity^34^ and level^34^, ^35^ of physical activity also decreases with age. Physical inactivity is associated with insulin resistance via several molecular mechanisms, including beta cells' insufficiency and inflammation.^36^ A previous study showed that high physical activity was associated with a low risk of diabetes mellitus in middle‐aged and older adults.^37^ Additionally, psychological changes, social changes, and multiple medications can be mentioned as other examples. One of the psychological changes with age is increasing depression symptoms.^38^ Tsai et al. showed that the presence of depression symptoms was associated with an increased risk of diabetes mellitus in older adults.^39^ Individuals with social engagement, such as living with someone and employment, which tend to decrease in older adults,^40^, ^41^ reportedly exhibit a low risk of diabetes mellitus.^42^ Older adults, particularly with age, have a higher rate of multiple medications,^43^ and that condition is associated with sarcopenia.^44^ Furthermore, survival bias may have existed regarding obesity and diabetes mellitus in older adults. These factors may account for the lack of a significant association between obesity and diabetes mellitus in women aged ≥75 years in the present study.

There are some potential reasons why sex differences were observed in the association between obesity and diabetes mellitus. First, the insulin sensitivity is different between men and women with obesity. A previous study involving Asian individuals aged 30–70 years showed that decreasing insulin sensitivity associated with obesity was more pronounced in men than in women.^45^ Second, other factors which may be associated with diabetes exist more commonly in women. For example, the study by Shimokata et al. indicated that muscle mass was lower in women than in men,^46^ and the National Health and Nutrition Survey in Japan reported that the proportions of individuals who were underweight and had undernutrition were higher in women.^10^ Psychogeriatric disorders^47^ and living alone^41^ were more common in older women. The sex differences observed in this study are likely to be influenced by these factors.

This study had some limitations. First, this study had a cross‐sectional design. Therefore, the causal relationship between obesity and metabolic diseases were unable to be clarified. However, several previous studies suggest that obesity precedes metabolic diseases,^16^, ^17^, ^48^, ^49^, ^50^ and it is reasonable to consider that this study is similar to these prior studies. Moreover, the health checkup data used in this study were obtained from a single center in Kyoto. As any individual who undergoes health checkups tends to be relatively healthy^51^ and has a good perceived health status,^52^ generalizing the results of this study to the entire Japanese population is difficult. Future studies are required to obtain data from multiple centers in Japan and clarify causality with a longitudinal design. Nevertheless, the strength of this study lies in the fact that the association of BMI and WC with metabolic diseases was examined across five age groups (18–44, 45–54, 55–64, 65–74, and ≥75 years), using a large health checkup data. The results of this study are thus important in assessing obesity for early prevention and control of metabolic diseases.

In conclusion, the present study indicated that individuals with obesity, based on BMI and WC, consistently had a high risk of hypertension, diabetes mellitus, and dyslipidemia among those aged 18–74 years, regardless of sex. Among those aged ≥75 years, obesity based on BMI and WC was associated with a high risk of metabolic diseases in men, but was associated with only hypertension and dyslipidemia in women. These findings suggest that the diagnosis criteria for obesity using BMI and WC could be applied to individuals aged <75 years, regardless of sex, and also to men aged ≥75 years.

## AUTHOR CONTRIBUTIONS

Writing ‐ review & editing: Yuiko Yamamoto and Kojiro Ishii; Writing – original draft: Yuiko Yamamoto, Noriko Satoh‐Asahara, Izuru Masuda, and Kojiro Ishii; Data acquisition: Megumi Kanasaki and Izuru Masuda; Data interpretation: Yuiko Yamamoto, Kentaro Ikeue, Hajime Yamakage, Noriko Satoh‐Asahara, Izuru Masuda, and Kojiro Ishii; Statistical analysis: Yuiko Yamamoto. All authors contributed to the article and approved the final version.

## CONFLICT OF INTEREST STATEMENT

The authors declare no conflicts of interest.

## Data Availability

The datasets used and/or analyzed during the current study are available from the corresponding author on reasonable request.
